
JOURNAL INFORMATION
==============================
NLM Title Abbreviation: Biospektrum (Heidelb)
Journal ID: Biospektrum (Heidelb)
NLM Journal ID: 9615685

Biospektrum

ISSN: 0947-0867
EISSN: 1868-6249

ARTICLE INFORMATION
==============================
PMCID: PMC8501349
PMID: 34658537
DOI: 10.1007/s12268-021-1642-0
Article ID: 1642
Article version: 1
Subjects: Wissenschaft

Campylobacteriose — eine zoonotische Infektionskrankheit

Alter Thomas 1
Bereswill Stefan 2
Backert Steffen steffen.backert@fau.de
3
1 grid.14095.39 0000 0000 9116 4836 Institut für Lebensmittelsicherheit und -Hygiene, FU Berlin, Berlin, Deutschland
2 grid.6363.0 0000 0001 2218 4662 Institut für Mikrobiologie und Infektionsimmunologie, Charité-Universitätsmedizin Berlin, Berlin, Deutschland
3 grid.5330.5 0000 0001 2107 3311 Department Biologie Lehrstuhl für Mikrobiologie, Friedrich-Alexander-Universität Erlangen-Nürnberg, Staudtstraße 5, D-91058 Erlangen, Deutschland
Electronic publication date: 2021 Oct 9
Print publication date: 2021
Volume: 27
Issue: 6
First page: 591
Last page: 593
Copyright: © Die Autoren 2021
License: Dieser Artikel wird unter der Creative Commons Namensnennung 4.0 International Lizenz veröffentlicht, welche die Nutzung, Vervielfältigung, Bearbeitung, Verbreitung und Wiedergabe in jeglichem Medium und Format erlaubt, sofern Sie den/die ursprünglichen Autor(en) und die Quelle ordnungsgemäß nennen, einen Link zur Creative Commons Lizenz beifügen und angeben, ob Änderungen vorgenommen wurden. Die in diesem Artikel enthaltenen Bilder und sonstiges Drittmaterial unterliegen ebenfalls der genannten Creative Commons Lizenz, sofern sich aus der Abbildungslegende nichts anderes ergibt. Sofern das betreffende Material nicht unter der genannten Creative Commons Lizenz steht und die betreffende Handlung nicht nach gesetzlichen Vorschriften erlaubt ist, ist für die oben aufgeführten Weiterverwendungen des Materials die Einwilligung des jeweiligen Rechteinhabers einzuholen. Weitere Details zur Lizenz entnehmen Sie bitte der Lizenzinformation auf http://creativecommons.org/licenses/by/4.0/deed.de.
License URL: https://creativecommons.org/licenses/by/4.0/

issue-copyright-statement: © The Author(s), under exclusive licence to Springer-Verlag GmbH Deutschland, ein Teil von Springer Nature 2021

==============================
Campylobacter jejuni represents an important zoonotic pathogen that is causing foodborne enteric infections. In the human gut, C. jejuni bacteria induce intestinal campylobacteriosis which can develop into systemic post-infectious sequelae such as Guillain-Barré syndrome or rheumatoid arthritis. Here, we review the pathobiology and molecular mechanisms of C. jejuni infections as well as promising strategies to combat campylobacteriosis within the “One World — One Health” approach.

■ Zoonosen sind bedeutende Infektionskrankheiten, die durch Viren, Bakterien, Pilze, Prionen sowie Parasiten zwischen Tieren und Menschen ausgetauscht werden. Frühere und aktuelle Ausbrüche von zoonotischen Viruserkrankungen, wie Ebola-Fieber, Zika-Fieber, Vogelgrippe und COVID-19, zeigen deutlich, wie eng zoonotische Krankheitserreger mit unserer Gesundheit verknüpft sind und uns bedrohen.

## *One World – One Health* bekämpft global Zoonosen

Etwa 60 Prozent aller Infektionskrankheiten werden ursprünglich von Tieren – oft über Lebensmittel – auf den Menschen übertragen. Die Statistiken der Weltgesundheitsorganisation WHO dokumentieren jährlich etwa 600 Millionen Krankheitsfälle und 420.000 Todesfälle, die durch kontaminierte Lebensmittel verursacht werden [1]. Daher ist ein breites Wissen über die Gesundheit und Krankheiten von Menschen, Haustieren und Wildtieren dringend erforderlich. Hierfür ist der *One World-One Health*-Ansatz besonders geeignet, um eine bessere Kontrolle von zoonotischen Infektionskrankheiten zu gewährleisten. Dieses Konzept basiert auf der Vorstellung, dass zoonotische Erkrankungen durch interdisziplinäre Kooperationen von Veterinär- und Humanmedizin unter Einbeziehung der Ökosysteme erfolgreich bekämpft werden können [2].

## Lebensmittel als Überträger von *Campylobacter jejuni*

Die bakteriell bedingten zoonotischen Infektionskrankheiten im Darm des Menschen werden von Krankheitserregern der Gattungen *Salmonella* und *Campylobacter* dominiert. Obwohl die Zahl der weltweit gemeldeten Salmonellen-Fälle in den vergangenen Jahren gesunken ist, bereitet die konstant hohe Zahl von *Campylobacter*-Infektionen enorme Probleme. Allein für die EU werden die Kosten der Campylobacteriose auf jährlich etwa 2,4 Milliarden Euro geschätzt, für die USA liegen die Zahlen bei bis zu 6,2 Milliarden Dollar [3]. Das hauptverantwortliche Bakterium, *Campylobacter jejuni*, besiedelt meist asymptomatisch den Darmtrakt von Wildvögeln und landwirtschaftlich relevantem Geflügel, aber auch von Rindern, Schweinen sowie Heimtieren [4]. Während des Schlachtprozesses, vor allem bei Masthähnchen, kann es durch fäkale Kontaminationen der Schlachtkörper zur Übertragung von *C. jejuni* auf Fleisch kommen. Darüber hinaus kann Rohmilch die Ursache von humanen Erkrankungen sein [5].

Die Infektionsrouten und klinischen Manifestationen sind in **Abb. 1** dargestellt. Die Infektionsdosis ist mit wenigen Hundert Bakterien recht gering. *C. jejuni* besiedelt den unteren Dünndarm und den Dickdarm. Die

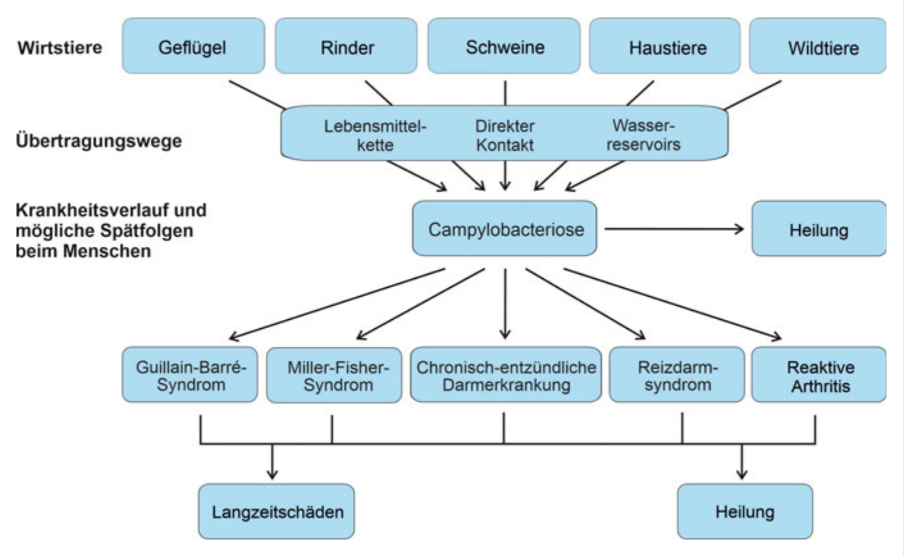

▲ **Abb. 1:** Natürliche Reservoirs, Übertragungswege und klinische Manifestationen von *Campylobacter jejuni* Infektionen. Weitere Details im Text.

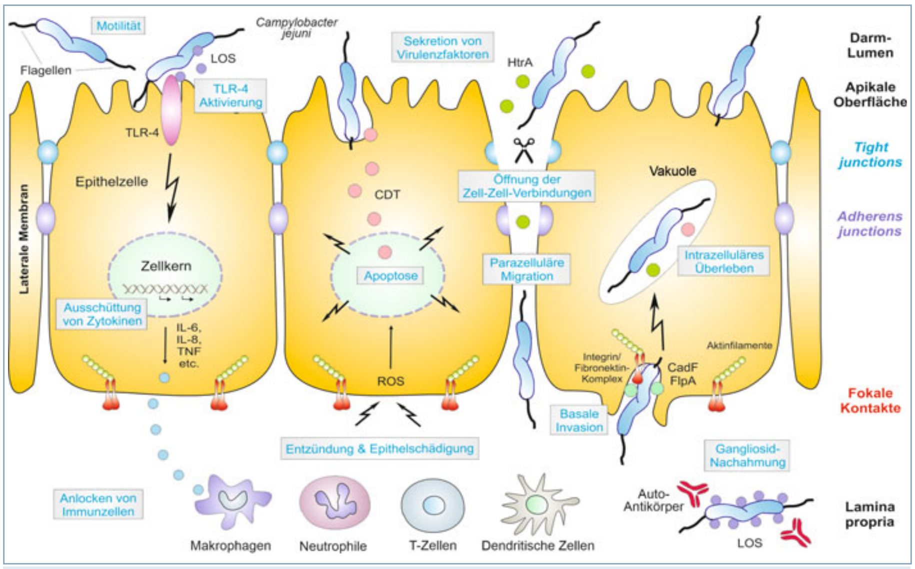

▲ **Abb. 2:** Molekulare Pathogenese-Mechanismen von *Campylobacter jejuni* im Darm. Das Darmepithel dient als physische Barriere und als Sensor für mikrobielle Infektionen wie durch *C. jejuni*. Verschiedene an der Oberfläche exponierte und sezernierte bakterielle Faktoren ermöglichen die *C. jejuni*-Kolonisierung, die Anheftung an Epithelzellen, die Öffnung der *tight* und *adherens junctions* (Sekretion von HtrA) und Transmigration sowie die basale Zellinvasion (Adhäsionsproteine CadF und FlpA) und das Überleben in intrazellulären Vakuolen. Hierbei erkennt TLR-4 das *C. jejuni*-Lipooligosaccharid (LOS). Von den Bakterien produzierte Faktoren induzieren nukleäre Reaktionen wie DNA-Schäden, Apoptose und die Produktion von pro-inflammatorischen Zytokinen. Letzteres führt zur Infiltration verschiedener Arten von Immunzellen an den Infektionsherd. Die Produktion reaktiver Sauerstoffspezies (ROS) sowie andere antibakterielle Reaktionen und das bakterielle Toxin CDT verstärken die Zellschädigung, was Campylobacteriose und schließlich das Absterben der Bakterien auslöst. Darüber hinaus ahmen modifizierte LOS-Strukturen von *C. jejuni* menschliche Ganglioside nach, was bei einigen Patient:innen zur Bildung von Autoantikörpern und zu neuronalen Störungen führen kann. Adaptiert mit Erlaubnis von [7]. Weitere Details im Text.

Bakterien dringen dort in die Epithelschicht ein und verursachen akute Entzündungsprozesse, da sie das angeborene Immunsystem aktivieren. Der Krankheitsverlauf ist somit vom Immunstatus des Wirts abhängig und variiert von leichten, schwach-entzündlichen, selbstlimitierenden Symptomen bis hin zu schweren, hoch-entzündlichen, blutigen Durchfällen, die mit starken Bauchschmerzen einhergehen und länger als eine Woche andauern [3,6].

In seltenen Fällen lösen *C. jejuni*-Infektionen immunologisch vermittelte postinfektiöse Erkrankungen mit autoimmuner Komponente aus. Dazu gehören neurologische Erkrankungen wie das Guillain-Barré-Syndrom und seine Variante, das Miller-Fisher-Syndrom, sowie Gelenksentzündungen, die Wochen nach der gastrointestinalen Manifestation auftreten (**Abb. 1**). Außerdem können *C. jejuni*-Infektionen auch zur Entwicklung eines Reizdarmsyndroms führen und Schübe einer chronisch-entzündlichen Darmerkrankung wie Morbus Crohn oder Colitis ulzerosa auslösen (**Abb. 1**, [3,6]).

Obwohl eine antimikrobielle Behandlung die Campylobacteriose um ein oder zwei Tage verkürzen kann, wird eine initiale Anwendung von Antibiotika nicht empfohlen [3,4]. Durch die hohen Resistenzraten von *C. jejuni* gegenüber Antibiotika aus den Gruppen der Makrolide und Fluorchinolone sowie die erschwerte Kontrolle der Antibiotikakonzentrationen bei selbstlimitierenden Durchfallepisoden sind Antibiotika kontraindiziert und für die Behandlung von schweren Verläufen oder vorerkrankten Patient:innen reserviert. Infolgedessen erhalten die meisten Erkrankten keine kausale medizinische Behandlung, sondern werden mit Maßnahmen gegen die Symptome – wie Rehydrierung und Substitution von Elektrolyten – versorgt.

## Molekulare Konzepte der Pathogenese und Virulenzfaktoren

Unser Wissen über molekulare Pathogenesemechanismen von *C. jejuni* beruht auf klinischen Studien und der Untersuchung von Bakterien in Zellkulturen *in vitro* sowie im Tiermodell (**Abb. 2**). Die Campylobacteriose ist durch eine Zerstörung des intestinalen Epithelgewebes gekennzeichnet, die im Rahmen der Entzündung von Immunzellen verursacht wird [7]. Die eindringenden Bakterien bewegen sich sehr effektiv mithilfe ihrer Flagellen und präsentieren dem Immunsystem das Endotoxin Lipooligosaccharid (LOS), das die akute Entzündung auslöst. LOS ist ein Glykolipid aus einem Oligosaccharid-Anteil und einem Lipid-A-Kern, das vom angeborenen Immunsystem durch den Toll-Like-Rezeptor 4 (TLR-4) erkannt wird [3]. Als Reaktion auf LOS locken dendritische Zellen und Makrophagen neutrophile Granulozyten an, die toxische Sauerstoffradikale (ROS) sowie Entzündungsmediatoren produzieren und eine Epithelzerstörung durch Apoptoseinduktion verursachen (**Abb. 2**). Diese Prozesse können durch das sekretierte Toxin CDT verstärkt werden [8], das von Teilpopulationen des Pathogens produziert wird. Die Apoptose resultiert in einer ulzerativen Gewebszerstörung, die eine (oft blutige) Diarrhö zur Folge hat. Von Makrophagen sezernierte Immunmediatoren wie Interleukine (IL) (z.B. IL-6, IL-8, IL-1β, IL-12 und IL-23) oder Tumornekrosefaktor (TNF) verstärken die Entzündungsreaktionen. Aktivierte T-Zellen produzieren neben entzündungsfördernden Interferonen (IFN), wie IFN-γ, IL-17 oder IL-22 auch anti-inflammatorische Zytokine wie z. B. IL-4 und IL-10, die die Immunantwort dämpfen und damit schweren Verläufen der Campylobacteriose entgegenwirken [3].

Einige *C. jejuni*-Stämme können das entzündliche Potenzial durch Sialylierung der LOS-Strukturen verstärken. Die unterschiedlichen Krankheitsverläufe können so auch durch die Variabilität des LOS der Bakterien erklärt werden. Die Sialylierung des Oligosaccharidanteils von LOS verstärkt zudem die Bindung an TLR-4 und damit den Zytokin-„Sturm", der die akute Entzündung vorantreibt [3]. Da sialylierte Oligosaccharidketten im LOS auch Gangliosiden im Nervensystem ähneln, induzieren individuelle *C. jejuni*-LOS-Strukturvarianten die Produktion von Anti-Gangliosid-Antikörpern, was in der Folge zu einer durch Makrophagen ausgelösten axonalen Destruktion und letzlich zum Guillain-Barré-Syndrom führen kann [3,8].

Über die Erforschung der Interaktionen von *C. jejuni* mit Darmepithelzellen identifizierten wir und andere Gruppen weitere bedeutsame Pathogenitätsmechanismen (**Abb. 2**). So befähigen Adhäsin-Proteine die

Bakterien zur Bindung an Enterozyten. Die Fibronektin-bindenden Proteine CadF und FlpA wurden im Detail untersucht [4,7,8]. Von der Unterseite der Zellen über einen basalen Mechanismus mittels Integrinrezeptoren vermitteln die genannten Proteine auch das Eindringen (Invasion) von *C. jejuni* in das intestinale Epithel. Die Fähigkeit zum intrazellulären Überleben in Vakuolen verstärkt die Pathogenität der Bakterien.

Darüber hinaus sekretiert *C. jejuni* die Serinprotease HtrA in das extrazelluläre Milieu. Diese Protease zerschneidet drei bedeutsame epitheliale Proteine: Occludin, Claudin-8 und E-cadherin [8]. Dies führt zu einer Öffnung der Zell-Zell-Verbindungen und Barriere-Dysfunktion. Die Bakterien sind somit in der Lage, die intestinale Epithelbarriere über die parazelluläre Route zu überwinden, gelangen so in tiefere Gewebsschichten und können im Rahmen von Bacteriämie und Sepsis sogar in andere Organe einwandern. Die gleichzeitig durch die Infektion gestörte Ionen-Homöostase im Darmepithel führt zum Verlust von Wasser und zu einer Diarrhö (Leckflux-Typ) [9]. Die erwähnten Immunmediatoren intensivieren zudem die Barriereschädigung. Sie verstärken durch Dysregulation des Natriumkanals ENaC auch die Diarrhö, die durch Natrium-Malabsorption gekennzeichnet ist [9].

*C. jejuni* ist ein weltweit verbreiteter Zoonoseerreger mit hoher Ansteckungsfähigkeit und -wahrscheinlichkeit, der aktuell noch nicht aus Lebensmittelketten eliminiert werden kann. Daher sollten zukünftige *One World-One Health*-basierte Kontrollmaßnahmen und Interventionsstrategien das Auftreten der Bakterien in Nutztieren minimieren und die Kontamination in der Lebensmittelproduktion reduzieren. So sollen neue technologische Dekontaminationsmaßnahmen dazu beitragen, die Hygiene auf Mast-, Schlacht- und Verarbeitungsebene zu verbessern [6]. Parallel muss das allgemeine Bewusstsein für lebensmittelassoziierte Krankheitserreger durch Informationskampagnen in der Bevölkerung weiter gefördert werden. Hier steht die Küchenhygiene bei der Verarbeitung von rohem Fleisch im Vordergrund. Die molekularen Mechanismen der Pathogenität werden darüber hinaus genutzt, um neue Therapeutika und Impfstoffe gegen die Campylobacteriose zu entwickeln. Aktuelle Strategien haben außerdem eine Entzündungshemmung zum Ziel, die auch vor Folgeerkrankungen wie dem Guillain-Barré-Syndrom oder verschiedenen chronischen Entzündungsprozessen schützen soll.


Funding note

Open Access funding enabled and organized by Projekt DEAL.

Danksagung

Diese Arbeit wurde vom Bundesministerium für Bildung und Forschung (BMBF) über die Projekte IP7/01KI2007D (SBe) IP9/01KI1725E (SBa) und IP2/01KI1725A (TA) im PAC-Campylobacter Forschungskonsortium gefördert. Wir danken Dr. N. Tegtmeyer für die Hilfe bei Abb. 1 und 2 und Prof. J.D. Schulzke für kritische Kommentare.

Thomas Alter 1989–1996 Studium der Veterinärmedizin an den Universitäten Leipzig und Dublin, Irland. Promotion und Habilitation an der Universität Leipzig, 2005–2009 Leiter des Nationalen Referenzlabors für Campylobacter am Bundesinstitut für Risikobewertung, Berlin. Seit 2009 geschäftsführender Direktor des Instituts für Lebensmittelsicherheit und -hygiene an der FU Berlin.

Stefan Bereswill 1985–1995 Biologiestudium mit Promotion an den Universitäten Kaiserslautern und Heidelberg, 1995–2003 AG-Leiter Helicobacter-Infektionen, Institut für Medizinische Mikrobiologie, Universität Freiburg, 2000 Habilitation an der Medizinischen Fakultät, Universität Freiburg. Seit 2003 Leiter AG Gastrointestinale Infektionen, Charité Berlin.

Steffen Backert 1987–1992 Biochemiestudium in Berlin. 1996 Promotion in Genetik an der HU Berlin. 1997–2003 AG-Leiter am Max-Planck-Institut für Infektionsbiologie, Berlin; 2003–2008 AG-Leiter und Habilitation an der Universität Magdeburg, 2008–2012 Professor für Zelluläre Mikrobiologie, University College Dublin, Irland. Seit 2013 Professor für Mikrobiologie an der Universität Erlangen.


Literatur

[1] WHO (2020) World Health Organisation. Campylobacter. www.who.int/news-rooni/fact-sheets/detail/campylobacter
[2] One World One Health (2021) One World — One Health™ — MISSION statement. https://oneworldonehealth.wcs.org/About-Us/Mission.aspx
[3] Heimesaat MM Backert S Alter T Human Campylobacteriosis-A Serious Infectious Threat in a One Health Perspective Curr Top Microbiol Immunol 2021 431 1 23 33620646 10.1007/978-3-030-65481-8_1
[4] Burnham PM Hendrixson DR Campylobacter jejuni: collective components promoting a successful enteric lifestyle Nat Rev Microbiol 2018 16 551 565 10.1038/s41579-018-0037-9 29892020
[5] RKI (2020) Infektionsepidemiologisches Jahrbuch. www.rki.de/DE/Content/Infekt/Jahrbuch/jahrbuch_node.html
[6] Alter T Reich F Management Strategies for Prevention of Campylobacter Infections Through the Poultry Food Chain: A European Perspective Curr Top Microbiol Immunol 2021 431 79 102 33620649 10.1007/978-3-030-65481-8_4
[7] Backert S Hofreuter D Molecular methods to investigate adhesion, transmigration, invasion and intracellular survival of the foodborne pathogen Campylobacter jejuni J Microbiol Meth 2013 95 8 23 10.1016/j.mimet.2013.06.031 23872466
[8] Tegtmeyer N Sharafutdinov I Harrer A Campylobacter Virulence Factors and Molecular Host-Pathogen Interactions Curr Top Microbiol Immunol 2021 431 169 202 33620652 10.1007/978-3-030-65481-8_7
[9] Lobo de Sá FD Schulzke JD Bücker R Diarrheal Mechanisms and the Role of Intestinal Barrier Dysfunction in Campylobacter Infections Curr Top Microbiol Immunol 2021 431 203 231 33620653 10.1007/978-3-030-65481-8_8
